# Assessing Chemical-Induced Liver Injury *In Vivo* From *In Vitro* Gene Expression Data in the Rat: The Case of Thioacetamide Toxicity

**DOI:** 10.3389/fgene.2019.01233

**Published:** 2019-11-26

**Authors:** Patric Schyman, Richard L. Printz, Shanea K. Estes, Tracy P. O’Brien, Masakazu Shiota, Anders Wallqvist

**Affiliations:** ^1^DoD Biotechnology High Performance Computing Software Applications Institute, Telemedicine and Advanced Technology Research Center, U.S. Army Medical Research and Development Command, Fort Detrick, MD, United States; ^2^The Henry M. Jackson Foundation for the Advancement of Military Medicine Inc. (HJF), Bethesda, MD, United States; ^3^Department of Molecular Physiology and Biophysics, Vanderbilt University School of Medicine, Nashville, TN, United States

**Keywords:** predictive toxicology, RNA-seq, thioacetamide, toxicogenomics, fibrosis, *in vitro-in vivo* correlations

## Abstract

Consumers are exposed to thousands of chemicals with potentially adverse health effects. However, these chemicals will never be tested for toxicity because of the immense resources needed for animal-based (*in vivo*) toxicological studies. Today, there are no viable *in vitro* alternatives to these types of animal studies. To develop an *in vitro* approach, we investigated whether we could predict *in vivo* organ injuries in rats with the use of RNA-seq data acquired from tissues early in the development of toxicant-induced injury, by comparing gene expression data from RNA isolated from these rat tissues with those obtained from *in vitro* exposure of primary liver and kidney cells. We collected RNA-seq data from the liver and kidney tissues of Sprague-Dawley rats 8 or 24 h after exposing them to vehicle (control), low (25 mg/kg), or high (100 mg/kg) doses of thioacetamide, a known liver toxicant that promotes fibrosis; we used these doses and exposure times to cause only mild toxicant-induced injury. For the *in vitro* study, we treated two cell types from Sprague-Dawley rats, primary hepatocytes (vehicle; low, 0.025 mM; or high, 0.125 mM dose), and renal tube epithelial cells (vehicle; low, 0.125 mM; or high, 0.500 mM) dose) with the thioacetamide metabolite, thioacetamide-S-oxide, selecting *in vitro* doses and exposure times to recreate the early-stage toxicant-induced injury model that we achieved *in vivo*. RNA-seq data were collected 9 or 24 h after application of vehicle or thioacetamide-S-oxide. We found that our modular approach for the analysis of gene expression data derived from *in vivo* RNA-seq strongly correlated (R^2^ > 0.6) with the *in vitro* results at two different dose levels of thioacetamide/thioacetamide-S-oxide after 24 h of exposure. The top-ranked liver injury modules *in vitro* correctly identified the ensuing development of liver fibrosis.

## Introduction

Thioacetamide was developed as an effective pesticide to control the decay of citrus fruits (Childs and Siegler, 1945), but was soon found to cause liver diseases (fibrosis and cirrhosis) and liver tumors (Fitzhugh and Nelson, 1948). It has been used extensively in animal studies (Ledda-Columbano et al., 1991; Li et al., 2002; Yeh et al., 2004; Okuyama et al., 2005; Dwivedi and Jena, 2018), largely for its ability to cause acute liver damage (Li et al., 2002; Okuyama et al., 2005). As consumers, we are exposed to thousands of chemicals with potentially adverse health effects. Yet, many of these chemicals will never be tested for toxicity as extensively as thioacetamide because of the immense resources needed for animal-based (*in vivo*) toxicological studies. There is also an ethical aspect to conducting large-scale animal experiments, which must meet legal and regulatory requirements, as directed by the Animal Welfare Act (AWA) in the U.S. and the European Directive 2010/63/EU to implement the principles of replacement, reduction, and refinement of the use of animals in research. Consequently, major efforts are currently under way to develop non-animal–based testing methods, such as high-throughput cell-based (*in vitro*) assays (Dix et al., 2007; Adeleye et al., 2015; Goh et al., 2015; Wetmore, 2015). However, these platforms are unlikely to meet the requirements for safety assessment and replace animal testing until reliable *in vivo-in vitro* correlations are achieved (Wetmore, 2015). Therefore, we need an effective approach to link *in vitro* results to meaningful *in vivo* injury endpoints, such as liver fibrosis.

Here, we present our efforts to use a systems toxicology approach to address the discrepancies commonly found between *in vitro* and *in vivo* results. We focus on toxicogenomics, a subfield of toxicology, which assumes that toxicity is accompanied by a change in the expression of either a single gene or a set of genes (Hamadeh et al., 2002; Sutherland et al., 2017), and that chemical exposures leading to the same injury endpoint cause similar changes in gene expression. Toxicogenomics has advanced the understanding of toxicological effects and improved predictions of responses to chemicals (Steiner et al., 2004; Sutherland et al., 2017). Several toxicogenomic approaches use single genes or coexpressed genes to study stress responses. For example, when specific genes are associated with an injury (e.g., cancer, cholestasis, steatosis), gene signatures are often used to classify chemicals in terms of their toxicity endpoints (Segal et al., 2004; Sahini et al., 2014; Parmentier et al., 2017). When sets of genes are differentially activated in response to an injury condition, such gene sets are often referred to as toxicity pathways or coexpressed genes. Furthermore, data mining techniques, such as bi-clustering (Pontes et al., 2015), are used to create sets of coexpressed genes, which consist of genes whose expression pattern is correlated across a set of chemical exposures with a common injury endpoint (Sutherland et al., 2017).

Previously, we developed an unbiased protocol to assign sets of coexpressed genes (modules) associated with molecular toxicity and linked them to specific injuries in the liver and kidney, using the Iterative Signature Algorithm (Bergmann et al., 2003; Tawa et al., 2014; AbdulHameed et al., 2016; Te et al., 2016). Compared to gene signatures, coexpressed gene modules can make more robust predictions for specific pathologies because they rely on groups of genes rather than individual genes. Gene expression data are prone to inherent noise, owing to limitations in experimental techniques and the complexity of biological systems. A limitation of the gene module approach is that the modules do not contain the same mechanistic information as do biological pathways, where genes can be linked by their function. Using gene expression data from the Open Toxicogenomics Project-Genomics Assisted Toxicity Evaluation System (TG-GATEs) database, which contains data from Sprague-Dawley rats exposed to different chemicals for 4 to 29 days (Igarashi et al., 2015), we derived 8 and 11 chemical-induced injury modules for the liver and kidney, respectively, associated with the relevant histopathological injury phenotypes from the TG-GATEs database (Te et al., 2016). Recently, we validated our injury modules *in vivo* by exposing Sprague-Dawley rats to a low (25 mg/kg) or high (100 mg/kg) dose of thioacetamide for 8 or 24 h (Schyman et al., 2018). The most activated injury modules were those associated with cellular infiltration and fibrosis, consistent with previous studies on thioacetamide toxicity and suggested by our own histological analyses.

Our aim in this study was to test the hypothesis that injury modules identified from *in vitro* transcriptomic responses can correlate with injury modules from *in vivo* to assist in the prediction of *in vivo* injury endpoints. For the *in vitro*-*in vivo* comparison, we selected thioacetamide as a toxicant for its ability to cause acute liver damage (Li et al., 2002; Okuyama et al., 2005). Thioacetamide is highly toxic to the liver *in vivo* because it is rapidly metabolized by cytochrome P450 and flavin-containing monooxygenases into its reactive metabolites (thioacetamide-S-oxide and reactive oxygen species) (Hajovsky et al., 2012). In our *in vitro* studies, we used the metabolized form of the compound, thioacetamide-S-oxide (Hajovsky et al., 2012), to simulate the level of toxicity achieved *in vivo* after exposure to thioacetamide. To compare liver- and kidney-specific responses, we treated two types of primary cells from Sprague-Dawley rats, hepatocytes, and renal tube epithelial cells, with vehicle (control) or two different doses of thioacetamide-S-oxide (designated low and high), and collected RNA samples at two different time points to ensure a match to an *in vivo* early-stage injury model. For the *in vivo* comparisons, we used data from our published thioacetamide toxicity study (Schyman et al., 2018) of 30 Sprague-Dawley rats treated with either vehicle (control) or one of two doses (low; 25 mg/kg or high; 100 mg/kg) of thioacetamide to produce different degrees of early injury. We collected RNA samples for gene expression analysis from the liver and kidney 8 and 24 h following injection of vehicle or thioacetamide.

## Materials and Methods

### Experimental Procedures

#### Thioacetamide ***In Vivo***

Male Sprague-Dawley rats at 10 weeks of age were purchased from Charles River Laboratories (Wilmington, MA). Rats were fed with Formulab Diet 5001 (Purina LabDiet; Purina Miles, Richmond, IN) and given water *ad libitum* in an environmentally controlled room at a temperature of 23°C with a 12:12-h light-dark cycle. All experiments were conducted in accordance with the *Guide for the Care and Use of laboratory Animals* of the U.S. Department of Agriculture, the Vanderbilt University Institutional Animal Care and Use Committee, and USAMRDC Animal Care and Use Review Office. Animals (30 rats) were administered either vehicle (saline; 3 ml/kg; n = 5 each at two time points) or thioacetamide (25 or 100 mg/kg; n = 5 each at two time points for each dose) intraperitoneally at 9 am, and the liver and kidney from each animal were harvested 8 or 24 h after the administration of vehicle or thioacetamide. Rats were anesthetized by intravenous injection of sodium pentobarbital through a jugular vein catheter and then the liver and kidney were dissected and frozen using Wollenberger tongs precooled in liquid nitrogen. The collected plasma, urine, and organs were kept at -80°C until used for analyses. Frozen whole kidneys were powdered in liquid nitrogen, since this organ is histologically heterogeneous. Total RNA was isolated from the liver and powered kidney, using TRIzol Reagent (Thermo Fisher Scientific, Waltham, MA) and the direct-zol RNA MiniPrep kit (Zymo Research, Irvine, CA). We refer the reader to our original publication for further details (Schyman et al., 2018).

#### Thioacetamide ***In Vitro***

Cryopreserved rat (Sprague-Dawley) hepatocytes and renal proximal tubular epithelial cells were purchased from Triangle Research Labs (Research Triangle Park, NC) and Sciencell Research Laboratories (Carlsbad, CA), respectively. Hepatocytes were thawed and suspended in thawing medium (MCRT50; Triangle Research Labs) at 6–7 million cells/50 ml. The suspension was centrifuged at 50 ×g, and cells were resuspended in plating medium (MP100; Triangle Research Labs). Hepatocytes were plated on collagen 1-coated 96-well plates at a density of 2 × 10^4^ cells/well for measurement of cell viability and on collagen 1-coated 6-well plates at a density of 4.5 × 10^5^ cells/well for RNA sequence analysis. Cells were cultured under 5% CO_2_ in a 37°C incubator. After 4 h of culture to allow cell attachment, the medium was replaced with hepatocyte maintenance medium (CC-3198, Triangle Research Labs). Rat renal tubular epithelial cells were thawed and suspended in “Epithelial Cell Medium-animal” (EpiCM-a, Sciencell Research Laboratories) and plated into poly-L-lysine-coated 96-well plates at a density of 2 × 10^4^ cells/well for measurement of cell viability and on poly-L-lysine-coated 6-well plates at a density of 3 × 10^5^ cells/well for RNA collection. Cells were cultured under 5% CO_2_ in a 37°C incubator. After 4 h of culture to allow cell attachment, the medium was replaced by the same medium. Both rat hepatocytes and renal cells were cultured for an additional 18 h before addition of thioacetamide-S-oxide or vehicle (maintenance medium; CC-3198 for hepatocytes and EpiCM-a for renal cells).

Preliminary studies were performed on rat hepatocytes and renal cells to identify a dose (range: 0.025 to 4 mM for hepatocytes, 0.125 to 4 mM for renal cells) of thioacetamide-S-oxide and length of exposure (range: 3 to 24 h for both hepatocytes and renal cells) that would result in mild toxicity without substantial loss of cell viability. Two cell viability assays were performed. First, to measure cellular loss of lactate dehydrogenase (LDH), cells were collected and cellular LDH activity remaining after each treatment was measured using the Lactate Dehydrogenase Activity Assay Kit (Sigma-Aldrich, St. Louis, MO). Second, cellular adenosine triphosphate (ATP) levels were measured using the CellTier-Glo 2.0 Assay kit (Promega Co., Madison, WI) according to the manufacturer’s protocol. The time-course profiles of cell viablity in these prelimiary studies are illustrated in **Figures S1–S4** (**Supplemental Material**). Based on these studies, two time points, 9 and 24 h, and two doses of thioacetamide-S-oxide, designated low and high, were chosen to induce early-stage toxicant-nduced injury with no or little loss in cell viability. Hepatocytes and renal cells were thus exposed for 9 or 24 h to either vehicle or thioacetamide-S-oxide (0.025 or 0.125 mM for hepatocytes, and 0.125 or 0.5 mM for renal cells). **Table 1** shows the viability of hepatocytes and renal proximal tubular epithelial cells exposed to thioacetamide for 9 or 24 h compared to vehicle-exposed cells.

**Table 1:** Viability of hepatocytes and renal proximal tubular epithelial cells exposed to thioacetamide-S-oxide compared to vehicle-exposed cells at 9 or 24 h. The vehicle-exposed reference values for adenosine triphosphate (ATP) and lactate dehydrogenase (LDH) are relative values at each time point.

		9 h exposure		24 h exposure	
		ATP	LDH	ATP	LDH
Type of cells	Dose (mM)	%	%	%	%
Hepatocytes	0 (vehicle)	100 ± 3	100 ± 4	100 ± 3	100 ± 3
	0.025	99 ± 9	103 ± 6	84 ± 3	98 ± 7
	0.125	90 ± 5	99 ± 5	75 ± 6	95 ± 5
Epithelial Cells	0 (vehicle)	100 ± 2	100 ± 9	100 ± 1	100 ± 8
	0.125	99 ± 1	103 ± 11	91 ± 2	99 ± 6
	0.5	87 ± 6	95 ± 11	65 ± 5	90 ± 9

Total RNA was isolated from culture cells using TRIzol Reagent (Thermo Fisher Scientific) and the direct-zol RNA MiniPrep kit (Zymo Research). The isolated RNA samples were then submitted to the Vanderbilt University Medical Center VANTAGE Core (Nashville, TN) for RNA quality determination and sequencing. Total RNA quality was assessed using a 2100 Bioanalyzer (Agilent, Santa Clara, CA). At least 200 ng of DNase-treated total RNA with high RNA integrity was used to generate poly-A-enriched mRNA libraries, using KAPA Stranded mRNA sample kits with indexed adaptors (New England BioLabs, Ipswich, MA). Library quality was assessed using the 2100 Bioanalyzer (Agilent), and libraries were quantitated using KAPA library Quantification kits (KAPA Biosystems). Pooled libraries were subjected to 150-bp double-end sequencing using an Illumina NovaSeq6000 (San Diego, CA) according to the manufacturer’s protocol. Bcl2fastq2 Conversion Software (Illumina) was used to generate de-multiplexed Fastq files.

### Analysis of RNA-Seq Data

We used the RNA-seq data analysis tool Kallisto for read alignment and quantification (Bray et al., 2016). Kallisto pseudo-aligns the reads to a reference, producing a list of transcripts that are compatible with each read while avoiding alignment of individual bases. In this study, we pseudo-aligned the reads to the *Rattus norvegicus* transcriptome (Rnor_6.0) downloaded from the Ensembl website (Zerbino et al., 2018). Kallisto achieves a level of accuracy similar to that of other competing methods, but is orders of magnitude faster than alternative methods. Its speed allows for the use of a bootstrapping technique to calculate uncertainties of transcript abundance estimates by repeating the analyses after resampling with replacement. In this study, we employed this technique to repeat the analysis 100 times. The files from RNA-seq analysis were deposited in NCBI’s Gene Expression Omnibus (GEO) database under series accession numbers GSE120195 and GSE134569.

To identify differentially expressed genes (DEGs) from transcript abundance data, we used Kallisto’s companion analysis tool Sleuth, which uses the results of the bootstrap analysis during transcript quantification to directly estimate the technical gene variance for each sample (Pimentel et al., 2017). DEGs were defined by using a false discovery rate adjusted p-value (q-value) of no more than 0.05 and a minimum gene expression β-value of 0.41 as the criteria for differential expression, which corresponds to a fold-change (FC) value of 1.5. Note that the β-value is defined as the natural logarithm of the effect size, and that the effect size and FC value of a gene are not equivalent. Nonetheless, the ranking and the directionality of change in gene expression (i.e., if a gene is up- or down-regulated) should be the same. In the Supplemental Material, we provide the q-values of all genes and the DEGs.

### KEGG Pathway Analysis

To understand the biological significance of the alterations in gene expression levels induced by thioacetamide, we used the aggregated FC (AFC) method (Ackermann and Strimmer, 2009; Yu et al., 2017) to calculate significantly activated Kyoto Encyclopedia of Genes and Genomes (KEGG) pathways (Kanehisa and Goto, 2000). We downloaded the KEGG pathways from the Molecular Signatures Database (MsigDB) (c2.cp.kegg.v6.1.entrez.gmt) (Liberzon et al., 2011), provided by the Broad Institute, Cambridge, MA [http://software.broadinstitute.org/gsea/msigdb], in February 2018. This database contains pathway information curated from multiple online databases. Detailed descriptions and performance characteristics of the AFC method can be found in the original literature (Ackermann and Strimmer, 2009; Yu et al., 2017). In the AFC method, we first calculate the FC value for each gene (i.e., the difference between the mean log-transformed gene expression values for treatment and control conditions) and define the KEGG pathway score as the total FC value of all genes in the pathway. We then use the pathway scores to perform null hypothesis tests and estimate each pathway’s significance by its p-value, defined as the probability that the pathway score for a random data set is greater than the score from the actual pathway. The sign of the pathway score represents the direction of regulation: the pathway is defined as up-regulated if the gene expression level after a treatment condition is increased relative to the control condition, and down-regulated if it is instead reduced.

### Module Activation Score

We developed the aggregate absolute FC (AAFC) method to calculate the activation score of a gene set (Schyman et al., 2018). This method identifies gene sets (e.g., modules) that are significantly changed. The AAFC method first calculates the FC value for each gene, i.e., the difference between the mean log-transformed gene expression values for samples in the treatment and control cohorts. We assessed the significance of this FC value by Student’s t-test (n = 5 for both treatment and control cohorts). We updated the procedure from that in our previous study (Schyman et al., 2018), in which we used “qualified” genes that passed Student’s t-test (p-value < 0.05). In the updated version, we included all genes, but calculated a combined p-value for each gene set (module) using Fisher’s method as an indicator of robustness of the reliability of the genes in the module (Fisher, 1932). (See the Fisher values for all module calculations in **Tables S2–S5** of the Supplemental Material.) The AAFC method then calculated the absolute value of each gene’s log-transformed FC value, and for each gene set (e.g., module or pathway) calculated the total FC value of the absolute values. We then used the gene set scores to perform null hypothesis tests and estimated each gene set’s significance by its p-value, defined as the probability that the score for randomly selected FC values (10,000 times) is greater than the score from the actual gene set. A small p-value implies that the gene set value is significant. The z-score is the number of standard deviations by which the actual gene set value differs from the mean of the randomly selected FC values (10,000 times).

### Data Collection and Processing of High- and Low-Risk Liver Toxicants

For the *in vitro* assessment of high- and low-risk liver toxicity, we used data from TG-GATEs (Igarashi et al., 2015), a publicly available database that contains data associating chemical exposures with transcriptomic changes in the liver of male Sprague-Dawley rats. We processed the data according to our previous protocol (Tawa et al., 2014), using the ArrayQualityMetrics-Bioconductor package (Kauffmann et al., 2008) to assess the quality of the Robust Multiarray Averaged (RMA) preprocessed data (Irizarry et al., 2003). In this process, we removed outlier arrays and renormalized the remaining data.

For the *in vivo* data, we used DrugMatrix, a publically available toxicogenomics database. This database contains a large collection of gene expression data obtained from Sprague-Dawley rats after exposure to a range of chemicals (Ganter et al., 2005). This dataset utilizes the Affymetrix GeneChip Rat Genome 230 2.0 Array. We used the same protocol as described in our previous publication (AbdulHameed et al., 2014).

## Results

### Differentially Expressed Genes (DEGs) Activated *In Vitro* and *In Vivo*

**Table 2** summarizes the number of DEGs identified *in vivo* from liver and kidney exposed to thioacetamide treatment for 8 or 24 h and *in vitro* from primary hepatocytes and renal proximal tubular epithelial cells exposed to the thioacetamide metabolite, thiacetamide-S-oxide, for 9 or 24 h. The two time points and two doses of thioacetamide-S-oxide (see **Table 1**) produced minimal cytotoxicity, as indicated by two different cell viability assays. Thus, we attempted to mimic the level of toxicity induced by thioacetamide and its metabolites *in vivo* without attempting to match toxicant levels or time points exactly with those used previously for *in vitro* studies. The goal was to identify genes whose expression changes *in vivo* are associated with early injury induced by thioacetamide and/or its metabolites, which may also be altered similarly in rat primary liver and kidney cells following *in vitro* exposure to thioacetamide-S-oxide. In most cases, the number of DEGs identified in the liver and kidney depended on the dose (**Table 2**) with two exceptions: *in vitro* hepatocytes showed 4,292 DEGs 24 h after low-dose treatment compared to 3,178 DEGs 24 h after high-dose treatment, and *in vivo* kidneys showed 257 DEGs 8 h after low-dose treatment compared to 172 DEGs 8 h after high-dose treatment. Another notable trend in the *in vivo* study was that all kidney samples showed fewer DEGs than liver samples, consistent with the notion that the liver is responsible for metabolizing the majority of thioacetamide to reactive metabolites like thioacetamide-S-oxide. Thus, the liver was likely exposed to higher concentrations of this toxic thioacetamide metabolite, even though the *in vivo* dose of thioacetamide administered to the rats was the same for the liver and kidney. In contrast, rat liver and kidney primary cells were exposed *in vitro* directly to a high- and low-dose of the thioacetamide metabolite, thioacetamide-S-oxide. A high and low dose of thioacetamide-S-oxide was selected for each cell type to provide a similar level of cytotoxicity between each cell type (see **Table 1**). This led to a higher *in vitro* dose for renal tube epithelial cells than for hepatocytes, which might explain the liver and kidney discrepancy between the *in vivo* and *in vitro* results.

**Table 2:** Differentially expressed genes after exposure *in vivo* to thioacetamide and *in vitro* to thioacetamide-S-oxide.

	*In vivo*				*In vitro*			
	Low dose		High dose		Low dose		High dose	
	**8 h**	**24 h**	**8 h**	**24 h**	**9 h**	**24 h**	**9 h**	**24 h**
Liver	3027	1999	4443	4307	259	4292	2159	3178
Kidney	257	746	172	1571	890	71	2575	3529

**Figure 1:**
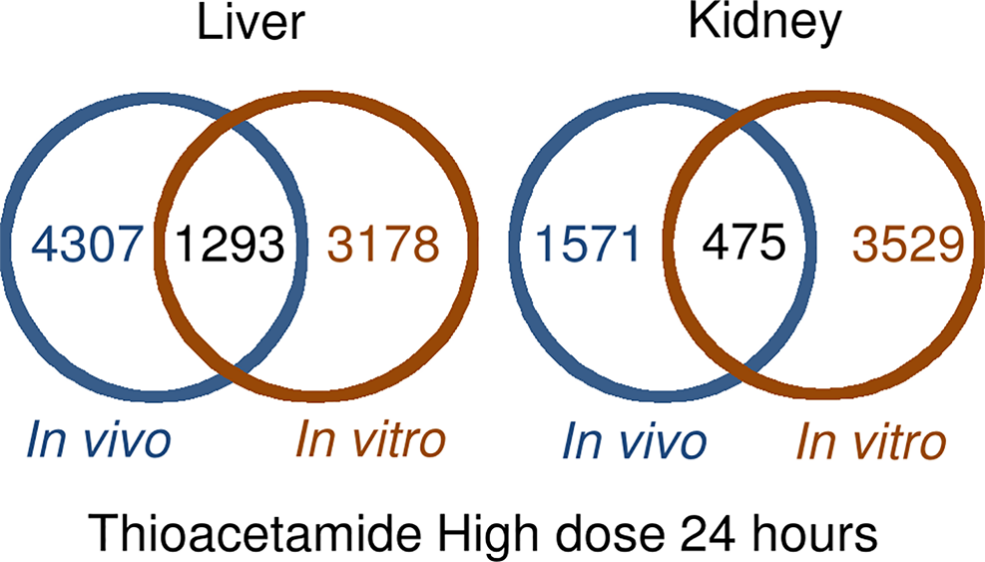
Overlap between differentially expressed genes in *in vivo* and *in vitro* 24 h after thioacetamide/thioacetamide-S-oxide exposure.

**Figure 2:**
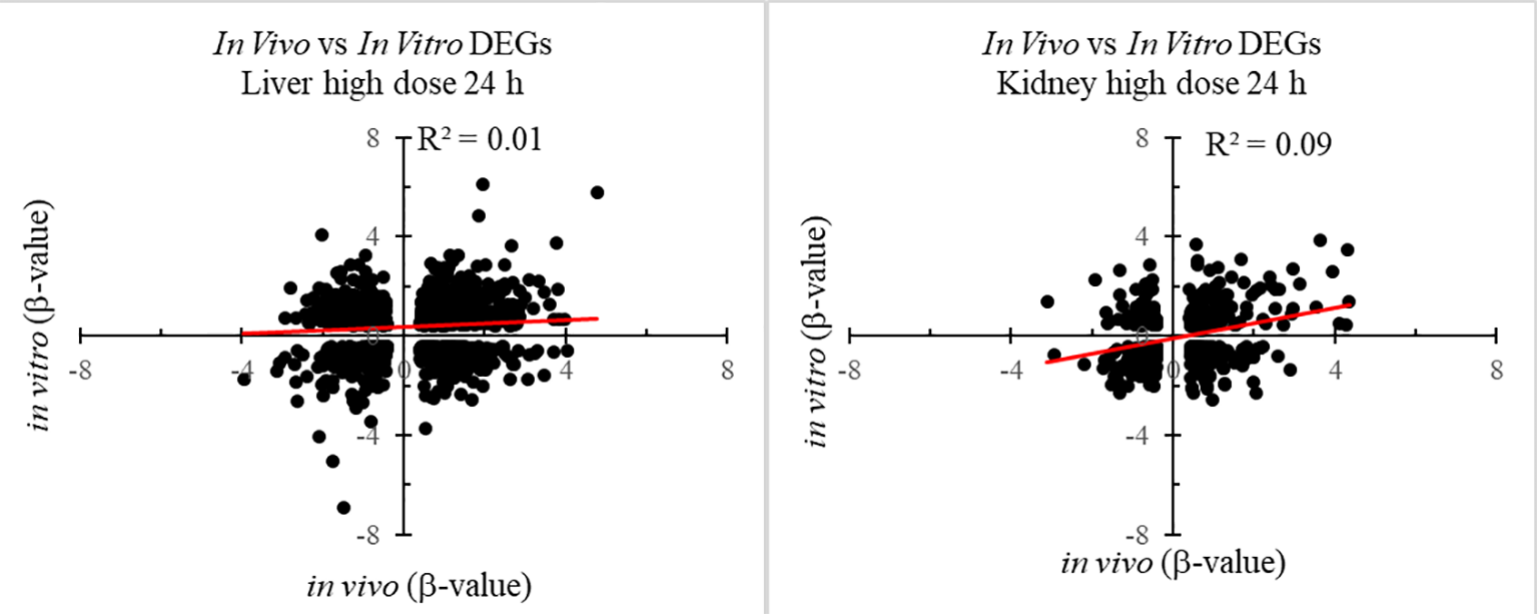
Correlation between differentially expressed genes (DEGs) in *in vitro* and *in vivo* 24 h after thioacetamide/thioacetamide-S-oxide exposure.

**Figure 1** shows the number of DEGs overlapping between those observed *in vivo* and *in vitro* for the liver and kidney 24 h after high-dose exposure to thioacetamide/thioacetamide-S-oxide. The overlap as a proportion of the total number of DEGs is small for the liver and kidney, covering approximately 20% and 10%, respectively. The overlapping genes might still be useful as predictors if they were correlated. However, an analysis of the relative gene expression changes *in vivo* and *in vitro* revealed no correlation for either the liver or kidney (**Figure 2**).

### KEGG Pathway Activation *In Vitro* and *In Vivo*

For our analysis, we used the KEGG pathway database (Kanehisa and Goto, 2000). To identify activated pathways we used the AFC method (Ackermann and Strimmer, 2009), which performs well compared to other popular pathway analysis methods (Yu et al., 2017). The AFC procedure uses all genes in a pathway to calculate the FC value and determine the significance by calculating the p-value using randomly selected genes (see *Materials and Methods* section).

**Table 3** summarizes the KEGG pathways that were significantly activated, with a p-value of < 0.05, both *in vivo* and *in vitro* for the liver and kidney 24 h after high-dose exposure to a form of thioacetamide. The *in vivo* and *in vitro* results showed overlap for 12 pathways in the liver and 13 pathways in the kidney. Changing the significance threshold to p < 0.01 resulted in only two overlapping pathways (*cardiac muscle contraction* and *oxidative phosphorylation*), which appeared both in the liver and the kidney. However, there were no overlapping pathways in the liver when we considered the directionality, whether a pathway was significantly overexpressed or suppressed (up- or down-regulated). **Table S1** in the **Supplemental Material** shows the KEGG pathways with their calculated p-values, 24 h after high-dose treatment *in vivo* and *in vitro* for both the liver and kidney.

**Table 3:** Overlap of Kyoto Encyclopedia of Genes and Genomes (KEGG) pathways significantly activated (p-value < 0.05) *in vivo* and *in vitro*, 24 h after high-dose thioacetamide/thioacetamide-S-oxide exposure.

KEGG pathway (p-value < 0.05, bold p-value < 0.01)	*In vivo-In vitro* overlap		Function
	Liver	Kidney	
Limonene and pinene degradation	√		Metabolism
PPAR signaling pathway	√		Endocrine system
Ascorbate and aldarate metabolism	√		Metabolism
Butanoate metabolism	√		Metabolism
Tyrosine metabolism	√		Metabolism
**Cardiac muscle contraction^a^**	**√**	**√**	**Circulatory system**
Antigen processing and presentation	√	√	Immune system
**Oxidative phosphorylation**	**√**	**√**	**Metabolism**
Glutathione metabolism	√	√	Metabolism
Linoleic acid metabolism	√	√	Metabolism
Metabolism of xenobiotics by cytochrome P450	√	√	Metabolism
Drug metabolism cytochrome P450	√	√	Metabolism
Pentose and glucuronate interconversions		√	Metabolism
Arginine and proline metabolism		√	Metabolism
Taurine and hypotaurine metabolism		√	Metabolism
Selenoamino acid metabolism		√	Metabolism
Porphyrin and chlorophyll metabolism		√	Metabolism
Nitrogen metabolism		√	Metabolism

^a^Bold text indicates significantly activated pathway with a p-value < 0.01.

### Injury Module Activation Analysis

We previously identified and evaluated 8 kidney and 11 liver injury modules (Te et al., 2016; Schyman et al., 2018). In those studies, we calculated the average absolute log_2_ FC value of all genes in a module to determine the activation score for each injury module. Here, we modified the procedure from our previous study (Schyman et al., 2018), where we only used genes that passed the t-test criteria, and instead included all genes. **Tables 4**–**7** show, in bold, the z-score values of significantly activated injury modules, for which the p-value was less than 0.01. **Tables S2–S5** in the Supplemental Material include the calculated p-values. For practical purposes, one should identify the top-ranking modules with the highest z-score values as the most likely injury phenotypes. However, several injury phenotypes may coexist, such as necrosis and cellular infiltration, which are early inflammatory responses.

**Table 4:** Activation of liver injury modules *in vivo* from rat liver tissue after exposure to thioacetamide.

Low dose				High dose			
8 h		24 h		8 h		24 h	
Module	z-score	Module	z-score	Module	z-score	Module	z-score
**Oval cell proliferation^a^**	**8.5**	**Anisonucleosis**	**14.7**	**Cellular infiltration**	**6.8**	**Cellular infiltration**	**14.9**
**Single cell necrosis**	**6.7**	**Cellular infiltration**	**14.2**	**Single cell necrosis**	**6.0**	**Fibrosis**	**12.0**
**Nuclear alteration**	**5.6**	**Fibrosis**	**11.1**	**Oval cell proliferation**	**4.6**	**Cellular foci**	**10.0**
**Cellular infiltration**	**5.2**	**Oval cell proliferation**	**9.6**	**Cellular foci**	**3.8**	**Anisonucleosis**	**9.1**
**Anisonucleosis**	**3.4**	**Cellular foci**	**9.0**	**Anisonucleosis**	**3.7**	**Oval cell proliferation**	**8.0**
**Bile duct proliferation**	**3.2**	**Single cell necrosis**	**4.6**	**Fibrosis**	**3.1**	**Single cell necrosis**	**6.5**
Cellular foci	1.8	**Nuclear alteration**	**4.1**	**Bile duct proliferation**	**3.1**	**Nuclear alteration**	**5.0**
Hematopoiesis	1.5	**Bile duct proliferation**	**3.6**	**Nuclear alteration**	**2.4**	**Bile duct proliferation**	**4.8**
Fibrosis	1.1	**Hematopoiesis**	**2.9**	**Hematopoiesis**	**2.3**	**Hematopoiesis**	**3.4**
Cytoplasmic alteration	0.8	Granular degeneration	1.4	Cytoplasmic alteration	1.1	Cytoplasmic alteration	-0.5
Granular degeneration	-0.8	Cytoplasmic alteration	0.2	Granular degeneration	0.2	Granular degeneration	-0.9

^a^Bold text indicates significantly activated module (p-value < 0.01).

**Table 5:** Activation of kidney injury modules *in vivo* from rat kidney tissue after exposure to thioacetamide.

Low dose				High dose			
8 h		24 h		8 h		24 h	
Module	z-score	Module	z-score	Module	z-score	Module	z-score
**Necrosis^a^**	5.2	Necrosis	2.6	**Necrosis**	7.3	**Necrosis**	**15.3**
Dilatation	0.6	Dilatation	1.6	Cellular infiltration	1.7	**Cellular infiltration**	**11.2**
Degeneration	0.2	Degeneration	0.9	Degeneration	1.5	**Degeneration**	**11.2**
Cellular infiltration	-1.8	Cellular infiltration	0.7	Hyaline cast	0.6	**Hyaline cast**	**6.4**
Hyaline cast	-2.0	Hyaline cast	-0.5	Hypertrophy	0.2	**Dilatation**	**6.4**
Hypertrophy	-2.1	Hypertrophy	-1.0	Intracytoplasmic inclusion body	-0.3	Fibrosis	1.5
Intracytoplasmic inclusion body	-2.4	Intracytoplasmic inclusion body	-2.7	Dilatation	-0.7	Intracytoplasmic inclusion body	-0.1
Fibrosis	-3.7	Fibrosis	-3.0	Fibrosis	-0.9	Hypertrophy	-1.7

^a^Bold text indicates significantly activated module (p-value < 0.01).

**Table 6:** Activation of liver injury modules *in vitro* from rat hepatocytes after exposure to the thioacetamide metabolite, thioacetamide-S-oxide.

Low dose				High dose			
9 h		24 h		9 h		24 h	
Module	z-score	Module	z-score	Module	z-score	Module	z-score
Hematopoiesis	-0.4	**Fibrosis^a^**	**6.4**	Hematopoiesis	1.0	**Anisonucleosis**	**6.9**
Anisonucleosis	-1.3	**Anisonucleosis**	**5.6**	Anisonucleosis	0.3	**Fibrosis**	**6.5**
Granular degeneration	-1.5	**Cellular foci**	**5.2**	Single cell necrosis	0.1	**Cellular foci**	**6.1**
Fibrosis	-1.8	**Bile duct proliferation**	**3.4**	Granular degeneration	-0.7	**Bile duct proliferation**	**3.7**
Bile duct proliferation	-1.8	**Cellular infiltration**	**3.3**	Fibrosis	-0.9	**Cellular infiltration**	**3.6**
Cytoplasmic alteration	-2.2	Single cell necrosis	2.4	Nuclear alteration	-0.9	Oval cell proliferation	1.2
Cellular infiltration	-2.2	Oval cell proliferation	2.2	Cellular foci	-1.1	Single cell necrosis	1.0
Cellular foci	-2.3	Nuclear alteration	0.2	Oval cell proliferation	-1.4	Nuclear alteration	0.1
Single cell necrosis	-2.4	Hematopoiesis	0.0	Bile duct proliferation	-2.1	Hematopoiesis	-0.2
Oval cell proliferation	-3.8	Granular degeneration	-1.6	Cellular infiltration	-2.1	Cytoplasmic alteration	-1.6
Nuclear alteration	-4.5	Cytoplasmic alteration	-1.6	Cytoplasmic alteration	-2.2	Granular degeneration	-1.9

^a^Bold text indicates significantly activated module (p-value < 0.01).

**Table 7:** Activation of kidney injury modules *in vitro* from renal tube epithelial cells after exposure to thioacetamide metabolite, thiacetamide-S-oxide.

Low dose				High dose			
9 h		24 h		9 h		24 h	
Module	z-score	Module	z-score	Module	z-score	Module	z-score
**Fibrosis^a^**	**3.3**	Hypertrophy	1.6	Hypertrophy	1.8	Cellular infiltration	1.9
Hypertrophy	1.7	Intracytoplasmic inclusion body	-0.2	Necrosis	1.3	Fibrosis	1.1
Cellular infiltration	1.3	Fibrosis	-0.3	Degeneration	1.2	Intracytoplasmic inclusion body	1.1
Degeneration	1.0	Cellular infiltration	-1.1	Dilatation	1.1	Hypertrophy	1.0
Dilatation	0.8	Dilatation	-1.1	Fibrosis	1.1	Necrosis	0.5
Intracytoplasmic inclusion body	0.0	Necrosis	-1.2	Cellular infiltration	0.4	Dilatation	0.1
Hyaline cast	-0.2	Hyaline cast	-1.3	Intracytoplasmic inclusion body	0.2	Hyaline cast	-0.5
Necrosis	-0.6	Degeneration	-1.8	Hyaline cast	-0.6	Degeneration	-1.2

^a^Bold text indicates significantly activated module (p-value < 0.01).

#### Liver Module Activation ***In Vivo***

In rats, thioacetamide exposure significantly activated (p-value < 0.01) several injury modules in the liver (**Table 4**). For each condition, the injury modules were ranked by the z-score from the most to the least likely injury endpoint. 8 h after thioacetamide exposure, all liver injury modules significantly activated at the low dose were also activated at the high dose, albeit with different ranking orders. Similarly, 24 h after low-dose exposure, all significantly activated injury modules were also activated after high-dose exposure. There was a significant overlap between the top-ranked liver injury modules at 24 h after low- and high-dose exposures. The difference in z-scores of the injury modules between 8 and 24 h were more pronounced than between low- and high-dose treatments.

#### Kidney Module Activation ***In Vivo***

Thioacetamide exposures significantly activated (p-value < 0.01) only a few injury modules in the kidney (**Table 5**). Necrosis was consistently the top-ranked kidney injury module in all kidney samples after thioacetamide exposure. At 24 h after high-dose exposure, several kidney injury modules were significantly activated, but necrosis was still the top-ranked injury phenotype.

#### Liver Module Activation ***In Vitro***

**Table 6** shows the liver injury modules activated by thioacetamide-S-oxide (the reactive metabolite of thioacetamide) in hepatocytes. 9 h after low- and high-dose treatments, no liver injury modules were significantly activated (p-value < 0.01). However, after 24 h, fibrosis was among the two highest-ranked injury modules for both low- and high-dose treatments. All five injury modules that were significantly activated 24 h after the low-dose treatment were also significantly activated after the high-dose treatment.

#### Kidney Module Activation ***In Vitro***

**Table 7** shows the kidney injury module activation scores for thioacetamide-S-oxide in renal proximal tubular epithelial cells. None of the high-dose treatments significantly activated any injury modules, but 9 h after exposure to the low-dose treatment the kidney injury module, fibrosis, was slightly activated. However, this injury module was not significantly (p-value < 0.01) activated at 24 h after treatment.

#### ***In Vitro***-***In Vivo*** Correlation

At 24 h after the high-dose treatment, three of the four liver injury modules that were ranked highest *in vitro* could be found among the four highest-ranked modules *in vivo* (**Tables 4** and **6**). Fibrosis was the second-highest ranked injury module *in vitro* and *in vivo*. These results are in agreement with fibrosis being the primary injury endpoint of thioacetamide exposure.

**Figure 3:**
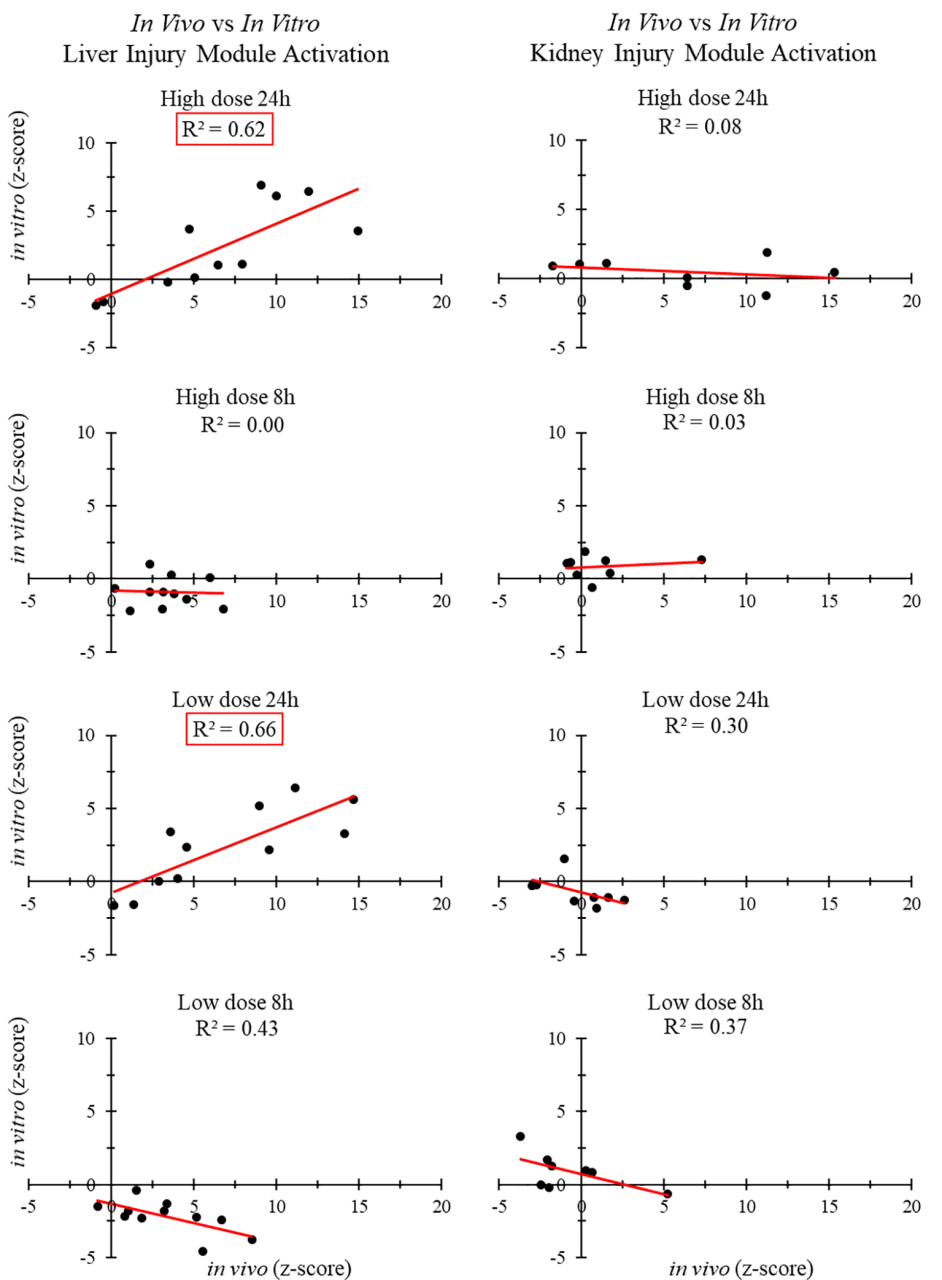
Correlation between injury modules activated *in vivo* and *in vitro*.

**Figure 3** shows the correlation between *in vivo* and *in vitro* experiments for different liver and kidney samples. The injury modules activated in the liver 24 h after high- and low-dose treatment were strongly correlated (R^2^ > 0.6). However, those activated in the kidney 24 h after high-dose and low-dose treatment showed no correlation. There was no positive *in vivo-in vitro* correlation at the shorter time points, regardless of the dose.

### *In Vitro* Predictions of High- and Low-Risk Liver Toxicants

In this section, we extend our validation to identify key injury phenotypes and to differentiate high-risk liver toxicants from low-risk toxicants. To this end, we identified four compounds with *in vitro* data from TG-GATEs and corresponding *in vivo* data from DrugMatrix. We selected carbon tetrachloride and lomustine as high-risk toxicants, which are known to promote fibrosis in rats after 29 days of exposure to 300 mg/kg of carbon tetrachloride and after 29 days of exposure to 6 mg/kg of lomustine (Igarashi et al., 2015). The doses used in DrugMatrix were similar: 400 mg/kg for carbon tetrachloride and 4.20 and 8.75 mg/kg for lomustine (Ganter et al., 2005). We classified naproxen and tamoxifen as low-risk toxicants, based on the LiverTox website (Hoofnagle et al., 2013), as liver fibrosis was not detected in the histology reports from TG-GATEs.

The *in vitro* data from TG-GATEs contain repeated-dose exposure to chemicals at low, medium, and high doses at three different time-points (2, 8, and 24 h). For this analysis, we calculated a module activation score for each condition and selected the maximum score. We provide all activation scores in **Table S6** (**Supplemental Material**).

**Table 8** shows the maximum injury module activation scores from *in vitro* and *in vivo* expression data for the four compounds. The fibrosis module was significantly activated for the two high-risk compounds in both *in vitro* and *in vivo*, but not for the low-risk compounds.

**Table 8:** Activation of liver injury modules *in vitro* and *in vivo* in rat hepatocytes after exposure to high- and low-risk liver toxicants. Bold text indicates significant activation of the Fibrosis module (p-value < 0.01).

High-risk liver toxicants							
Carbon tetrachloride				Lomustine			
*In vivo* (DrugMatrix)^a^		*In vivo* (TG-GATEs)^b^		*In vivo* (DrugMatrix)^c^		*In vitro* (TG-GATEs)^d^	
Low-risk liver toxicants							
Naproxen				Tamoxifen			
*In vivo* (DrugMatrix)^e^		*In vivo* (TG-GATEs)^f^		*In vivo* (DrugMatrix)^g^		*In vitro* (TG-GATEs)^h^	
Anisonucleosis	12.0	**Fibrosis**	**4.5**	**Fibrosis**	**10.2**	**Fibrosis**	**2.9**
**Fibrosis**	**11.1**	Anisonucleosis	4.4	Cellular infiltration	8.3	Anisonucleosis	2.4
Cellular infiltration	10.5	Hematopoiesis	3.7	Cellular foci	7.7	Cellular infiltration	2.2
Cellular foci	8.2	Cellular foci	2.8	Single cell necrosis	4.8	Granular degeneration	2.0
Single cell necrosis	5.9	Cellular infiltration	1.8	Hematopoiesis	4.5	Oval cell proliferation	1.9
Nuclear alteration	3.4	Nuclear alteration	1.7	Anisonucleosis	2.4	Bile duct proliferation	1.9
Hematopoiesis	2.2	Granular degeneration	1.7	Bile duct proliferation	2.2	Hematopoiesis	1.7
Oval cell proliferation	1.6	Single cell necrosis	1.3	Oval cell proliferation	2.0	Cellular foci	1.6
Granular degeneration	0.3	Cytoplasmic alteration	1.0	Cytoplasmic alteration	1.3	Cytoplasmic alteration	0.7
Cytoplasmic alteration	-0.1	Oval cell proliferation	0.8	Granular degeneration	0.9	Nuclear alteration	0.1
Bile duct proliferation	-0.3	Bile duct proliferation	0.7	Nuclear alteration	0.4	Single cell necrosis	-0.2
Nuclear alteration	0.3	Granular degeneration	4.5	Anisonucleosis	3.4	Anisonucleosis	4.6
Cytoplasmic alteration	0.0	Hematopoiesis	2.4	Granular degeneration	1.8	Hematopoiesis	2.7
Single cell necrosis	-0.3	Cytoplasmic alteration	1.9	Cytoplasmic alteration	1.1	Fibrosis	1.8
Oval cell proliferation	-0.5	Nuclear alteration	1.9	Nuclear alteration	0.2	Cytoplasmic alteration	1.7
Bile duct proliferation	-1.7	Bile duct proliferation	1.7	Single cell necrosis	0.1	Granular degeneration	1.6
Anisonucleosis	-2.1	Anisonucleosis	1.5	Fibrosis	-0.4	Oval cell proliferation	1.4
Cellular foci	-2.4	Oval cell proliferation	1.2	Cellular foci	-0.5	Nuclear alteration	1.4
Cellular infiltration	-2.6	Fibrosis	1.1	Cellular infiltration	-0.7	Cellular infiltration	1.0
Granular degeneration	-2.6	Single cell necrosis	0.9	Bile duct proliferation	-0.9	Cellular foci	0.8
Fibrosis	-2.9	Cellular foci	0.8	Hematopoiesis	-1.0	Single cell necrosis	0.1
Hematopoiesis	-3.9	Cellular infiltration	0.1	Oval cell proliferation	-1.0	Bile duct proliferation	-0.1

^a^Dose: 400 mg/kg; Time: 1, 3, 7, and 25 d. ^b^Dose: 1,000, 3,000, and 10,000 μM; Time: 2, 8, and 24 h. ^c^Dose: 4.2 and 8.75 mg/kg; Time: 1, 3, 5, and 25 d. ^d^Dose: 4.8, 24, and 120 μM; Time: 2, 8, and 24 h. ^e^Dose: 10 mg/kg; Time: 3 d. ^f^Dose: 80, 400, and 2000 μM; Time: 2, 8, and 24 h. ^g^Dose: 2.5 and 64 mg/kg; Time: 3 and 5 d. ^h^Dose: 0.12, 0.60, and 3.0 μM.

## Discussion

We used different approaches to assess the correlation between *in vitro* and *in vivo* toxicogenomic experiments. No positive correlations were observed for liver or kidney injury modules 8 or 9 h after thioacetamide/thioacetamide-S-oxide exposure. However, our injury module approach did indicate a strong correlation (R^2^ > 0.60) at 24 h after high-dose treatment of thioacetamide/thioacetamide-S-oxide between liver injury modules activated *in vitro* (in hepatocytes) and those activated *in vivo* (in rats), but no correlation (R^2^ < 0.10) between kidney injury modules activated *in vitro* (in renal cells) and those activated *in vivo* (in rats). Interestingly, there was also a strong *in vitro*-*in vivo* correlation (R^2^ = 0.66) for liver injury modules activated 24 h after low-dose treatment, which further indicates the sensitivity of the injury module approach to identifying specific injury phenotypes even after a low-dose exposure.

A noteworthy observation for the activation of liver injury modules *in vivo* and *in vitro* was the increase in activation score and rank of the fibrosis module over time (8 to 24 h) for both low- and high-dose treatments (see **Tables 4** and **6**). The opposite trend was observed for the single cell necrosis module *in vivo*, which was the top-ranked module 8 h after exposure but a mid-ranked module after 24 h (see **Table 4**). These observations are reasonable, given that fibrosis requires more time to develop than single cell necrosis with regard to an early immune response.

DEGs *in vitro* showed little overlap with DEGs *in vivo* (**Figure 1**), and gene FC values among the overlapping genes were uncorrelated (**Figure 2**). These data suggest that any *in vitro*-*in vivo* correlation based solely on individual genes are likely to be coincidental.

The KEGG pathway analysis of *in vivo* gene expression data from rats exposed to thioacetamide identified multiple biologically relevant pathways in the liver, as discussed previously (Schyman et al., 2018). In the *in vitro* hepatocyte experiment, 17 pathways (excluding human disease pathways) were significantly activated with a p-value of less than 0.05, which included 13 metabolic pathways, as well as cardiac muscle contraction, antigen processing and isoleucine biosynthesis, PPAR signaling, and complement and coagulation cascades pathways (see **Supplemental Material** for all activated KEGG pathways). Among the 17 pathways activated *in vitro*, 13 overlapped with the pathways activated in liver (*in vivo*) (**Table 3**). Nine of these pathways were related to metabolism (e.g., xenobiotic biodegradation and metabolism, carbohydrate metabolism, amino acid metabolism) and three others were related to the *circulatory*, *immune*, and *endocrine systems* (i.e., cardiac muscle contraction, antigen processing and isoleucine biosynthesis, and PPAR signaling pathways). Similarly, the majority of the significantly activated KEGG pathways in the kidney that showed *in vitro*-*in vivo* overlap were involved in metabolism (**Table 3**), and also *circulatory* and *immune systems* (i.e., cardiac muscle contraction and antigen processing and isoleucine biosynthesis). **Table 3** shows that more than half of the *in vitro*-*in vivo* overlapped pathways in liver or kidney also overlapped between liver and kidney samples. Some of these pathways were expected, such as metabolism of xenobiotics by cytochrome P450 and drug metabolism cytochrome p450, but also cardiac muscle contraction, antigen processing and isoleucine biosynthesis were common across organs. It is interesting to note that the glutathione metabolism pathway was also common, which is important in antioxidant defense and cellular function (e.g., cell proliferation, apoptosis, immune response) (Wu et al., 2004). However, these pathways, which are involved in general biological responses and are not injury specific, showed low specificity between the liver and kidney.

Previous *in vitro*-*in vivo* research has often focused on identifying correlated genes, pathways, or gene ontology terms (Zhang et al., 2013; De Abrew et al., 2015; Sutherland et al., 2016; Van den Hof et al., 2017; Taškova et al., 2018). We have shown that DEGs do not show satisfactory *in vitro*-*in vivo* correlations, which makes selection of individual genes indicative of injury unreliable. Furthermore, our KEGG pathway analysis identified several overlapping pathways between *in vivo* and *in vitro* conditions, but no clear link to fibrosis, the liver injury most associated with thioacetamide exposure.

Here, we show that our modular approach to identify gene sets specific to injury phenotypes performed well in assessing *in vivo* results from *in vitro* gene expression data after exposure to a thioacetamide metabolite, thioacetamide-S-oxide. An important factor in our modular approach is the use of absolute FC values to identify activated injury modules. Although this has been shown to be important in pathway analysis (Ackermann and Strimmer, 2009), it leads to loss of information about the directionality of change in the activated pathway. However, our results showed weak *in vivo*-*in vitro* correlations for the directionality of the FC (**Figure 2**), suggesting that the use of absolute FC values is critical to the success of the injury module approach.

Previous studies have typically focused on identifying gene signatures for a single injury phenotype (e.g., cancer, steatosis, cholestasis) (Segal et al., 2004; Sahini et al., 2014; Parmentier et al., 2017). Although this approach is adequate for understanding the underlying biological mechanisms of toxicity, a key aspect of predicting a specific organ injury phenotype is the ability to test and rank different injury phenotypes to identify the most likely injury. The prediction should also be organ-specific, which requires different sets of injury modules for different organs. Other studies have used similar multi-injury-gene set approaches to focus on liver injuries (De Abrew et al., 2015; Sutherland et al., 2017; Sutherland et al., 2019) or kidney injuries (Minowa et al., 2012).

A major limitation of our injury module approach is that we can only predict injuries for the injury modules we have identified. There are many more injury phenotypes for which we have not yet been able to assign a gene set, because publicly available data are still limited. Other limitations of our approach are that injury phenotypes are not necessarily indistinguishable, and that some injuries respond at different time scales (e.g., cellular infiltration is often an early response, whereas fibrosis is more pronounced later when the injury is more advanced). Additionally, our approach does not yet consider injury modules for other organs, such as the heart and brain.

**Figure 4:**
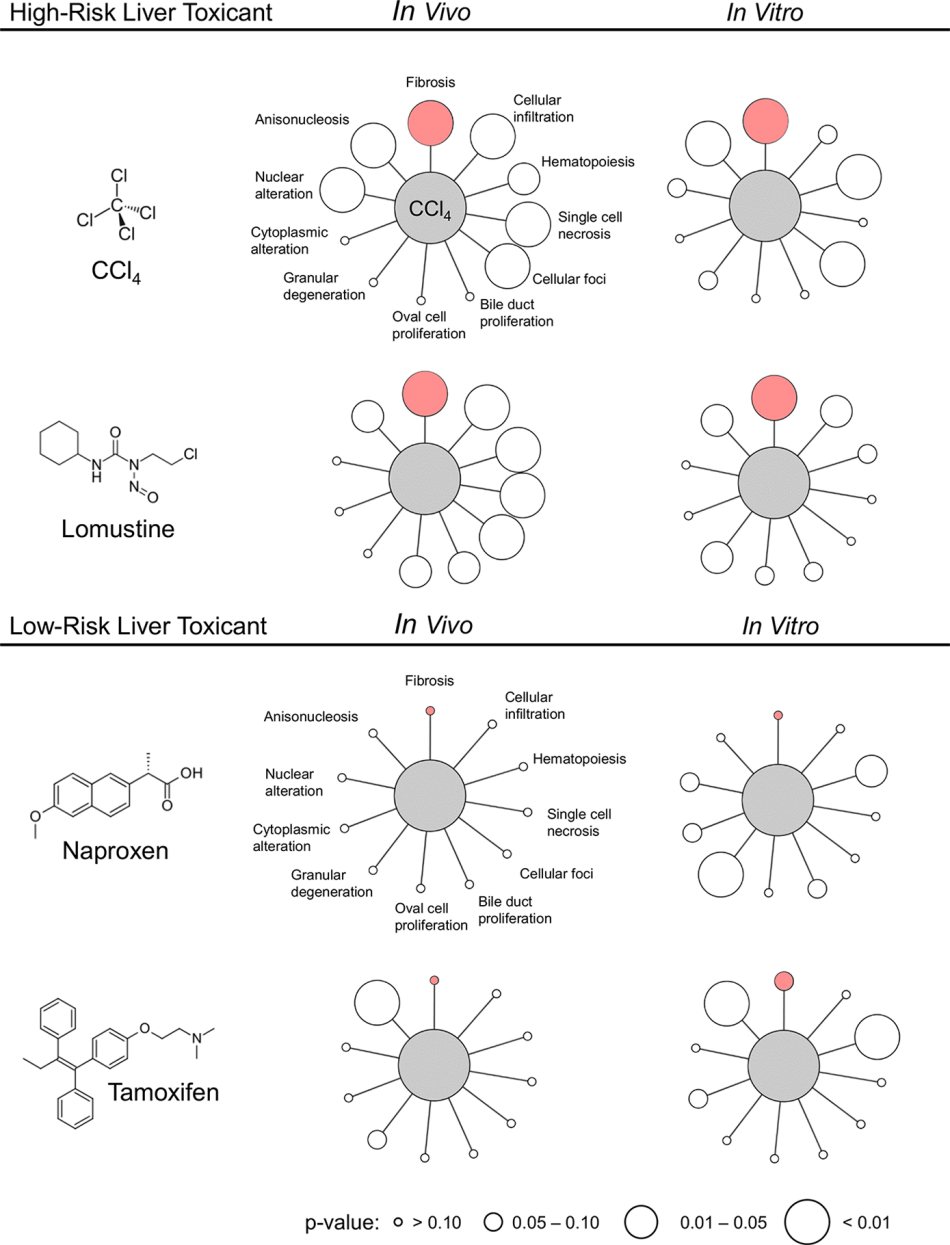
Activation of liver injury modules *in vitro* and *in vivo* in rat hepatocytes after exposure to high- and low-risk liver toxicants. Pink indicates the Fibrosis module and the size of the circle indicates the p-value. The image was generated using *Cytoscape* (Shannon et al., 2003).

To further validate the ability of our injury modules to predict *in vivo* injury endpoints, we selected four compounds with *in vitro* data from TG-GATEs and corresponding *in vivo* data in DrugMatrix. We did not expect perfect *in vitro*-*in vivo* concordance because the doses and time points were not necessarily determined in the same way (i.e., the highest dose with the least toxic response). However, our aim was to test whether we could identify a key injury phenotype and differentiate high-risk liver toxicants known to cause fibrosis from low-risk toxicants. **Figure 4** shows the significantly activated injury modules based on the activation scores in **Table 8**. For easy comparison we highlighted the fibrosis module in pink and the radius indicate the significance. We found that the injury module approach differentiated toxicants from non-toxicants based on activation scores and identified fibrosis as one of the injury phenotypes among the high-risk toxicants. Nontoxicants, when presented alone, only activated a few benign injury modules significantly.

In summary, our results support the hypothesis that coexpressed gene sets specific to an injury phenotype (injury modules) may be useful to predict *in vivo* injury endpoints, using RNA-seq data from *in vitro* cell studies. Although this method may never replace animal studies, in conjunction with other *in vitro* assays, it could facilitate the screening of large numbers of chemicals in order to predict liver and kidney injuries *in vivo*. Consequently, the approach can reduce the number of animals needed in experiments and improve the efficiency of toxicity assessments.

## Data Availability Statement

The files from RNA-seq analysis were deposited in NCBI’s Gene Expression Omnibus (GEO) database under series accession numbers GSE120195 and GSE134569.

## Ethics Statement

This study was carried out in accordance with the *Guide for the Care and Use of laboratory Animals* of the U.S. Department of Agriculture, using protocols approved by the Vanderbilt University Institutional Animal Care and Use Committee, and USAMRDC Animal Care and Use Review Office.

## Author Contributions

PS, RP, MS, and AW made substantial contributions to the concept and design of the work. MS designed the protocols for the animal studies. SE, TO’B, and MS performed the animal studies, analyzed injury markers in blood and urine, and collected samples. RP worked on the extraction and purification of RNA. PS analyzed the gene expression data and contributed to drafting the manuscript. RP, AW, and MS contributed to revising and editing the manuscript for important intellectual content.

## Funding

The authors were supported by the U.S. Army Medical Research and Development Command (Fort Detrick, MD), and the Defense Threat Reduction Agency grant CBCall14-CBS-05-2-0007.

## Conflict of Interest

The authors declare that the research was conducted in the absence of any commercial or financial relationships that could be construed as a potential conflict of interest.
