# Supplementary material for: Assessing Chemical-Induced Liver Injury In Vivo From In Vitro Gene Expression Data in the Rat: The Case of Thioacetamide Toxicity
Source: Front Genet. 2019 Nov 26;10:1233. doi: 10.3389/fgene.2019.01233 (PMC6901980; doi:10.3389/fgene.2019.01233)
Supplement: Supplementary file 1 [file Presentation_1.pptx]

## Slide 1
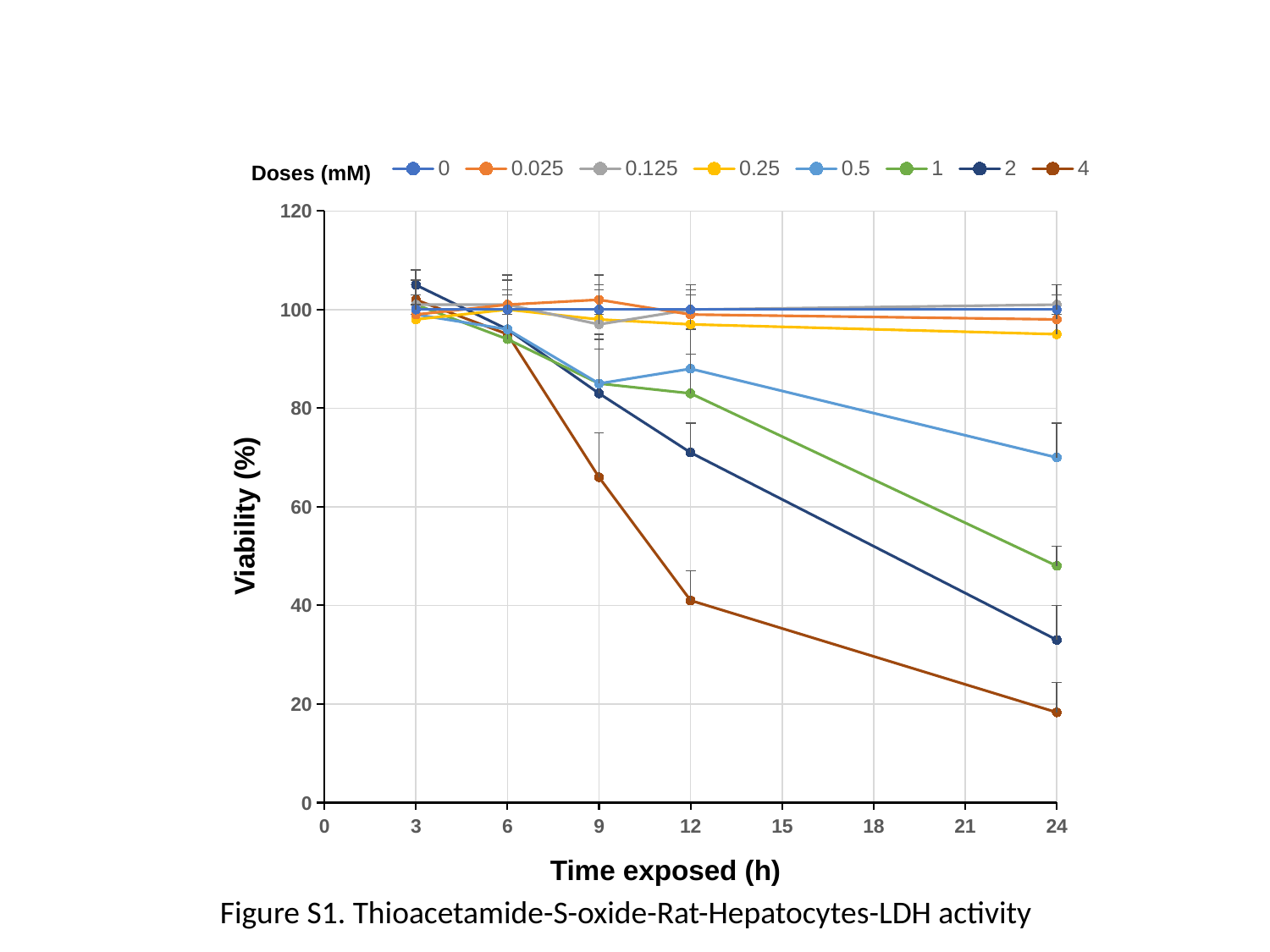

### Chart
| Category | 0 | 0.025 | 0.125 | 0.25 | 0.5 | 1 | 2 | 4 |
|---|---|---|---|---|---|---|---|---|Doses (mM)
Viability (%)
Time exposed (h)
Figure S1. Thioacetamide-S-oxide-Rat-Hepatocytes-LDH activity

## Slide 2
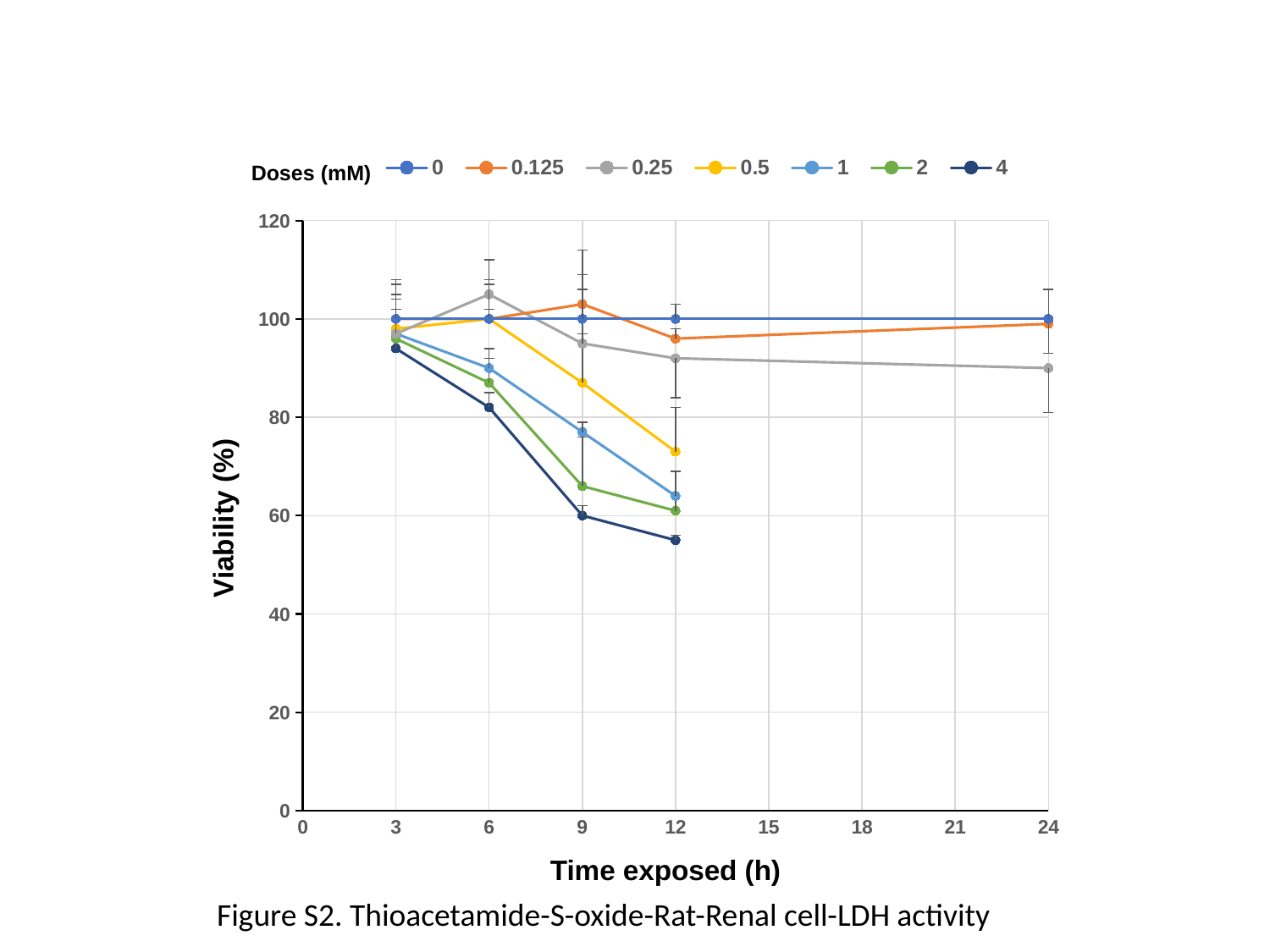

### Chart
| Category | 0 | 0.125 | 0.25 | 0.5 | 1 | 2 | 4 |
|---|---|---|---|---|---|---|---|Doses (mM)
Viability (%)
Time exposed (h)
Figure S2. Thioacetamide-S-oxide-Rat-Renal cell-LDH activity

## Slide 3
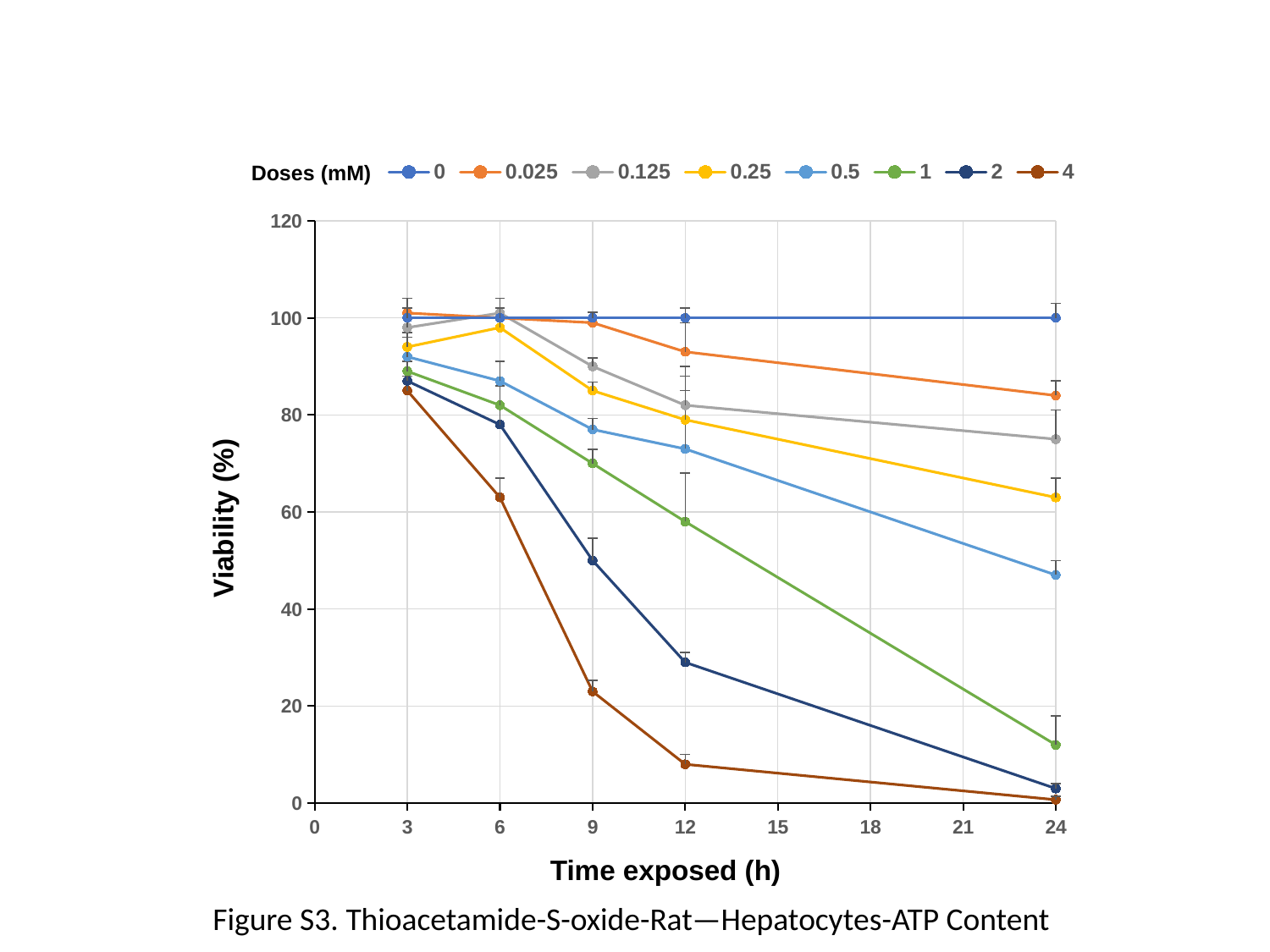

### Chart
| Category | 0 | 0.025 | 0.125 | 0.25 | 0.5 | 1 | 2 | 4 |
|---|---|---|---|---|---|---|---|---|Doses (mM)
Viability (%)
Time exposed (h)
Figure S3. Thioacetamide-S-oxide-Rat—Hepatocytes-ATP Content

## Slide 4
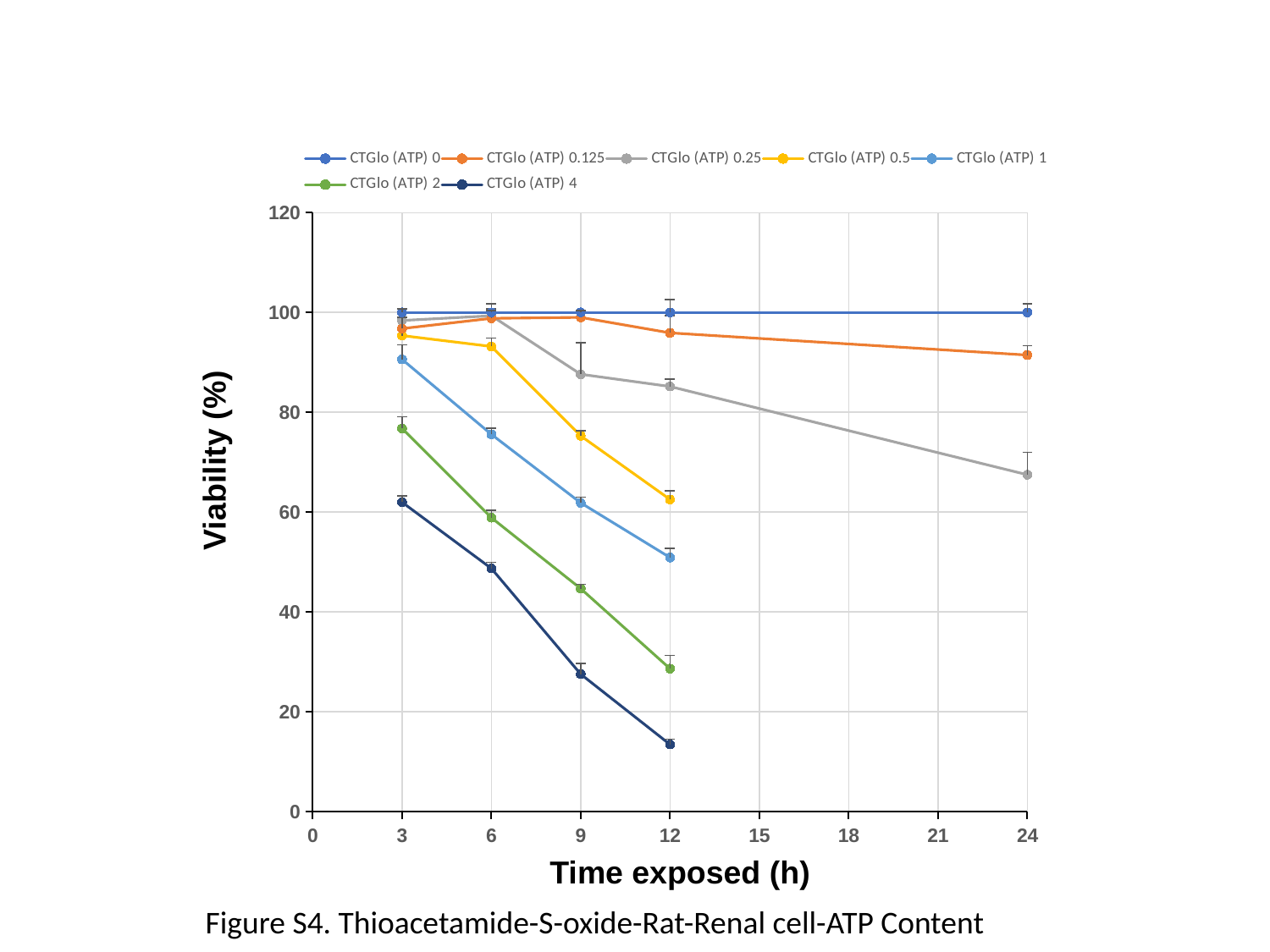

### Chart
| Category | CTGlo (ATP) 0 | CTGlo (ATP) 0.125 | CTGlo (ATP) 0.25 | CTGlo (ATP) 0.5 | CTGlo (ATP) 1 | CTGlo (ATP) 2 | CTGlo (ATP) 4 |
|---|---|---|---|---|---|---|---|Viability (%)
Time exposed (h)
Figure S4. Thioacetamide-S-oxide-Rat-Renal cell-ATP Content
